# Carcinoembryonic Cell Adhesion-Related Molecule 2 Regulates Insulin Secretion and Energy Balance

**DOI:** 10.3390/ijms20133231

**Published:** 2019-07-01

**Authors:** Elsaid Salaheldeen, Alexa Jaume, Sonia Michael Najjar

**Affiliations:** 1 Department of Biomedical Sciences, Heritage College of Osteopathic Medicine, Ohio University, Athens, OH 45701-2979, USA; 2 Department of Zoology, Faculty of Science, Sohag University, Sohag 82524, Egypt; 3 Diabetes Institute, Heritage College of Osteopathic Medicine, Ohio University, Athens, OH 45701-2979, USA

**Keywords:** hyperinsulinemia, insulin clearance, insulin resistance, hyperphagia, energy balance

## Abstract

The Carcinoembryonic Antigen-Related Cell Adhesion Molecule (CEACAM) family of proteins plays a significant role in regulating peripheral insulin action by participating in the regulation of insulin metabolism and energy balance. In light of their differential expression, CEACAM1 regulates chiefly insulin extraction, whereas CEACAM2 appears to play a more important role in regulating insulin secretion and overall energy balance, including food intake, energy expenditure and spontaneous physical activity. We will focus this review on the role of CEACAM2 in regulating insulin metabolism and energy balance with an overarching goal to emphasize the importance of the coordinated regulatory effect of these related plasma membrane glycoproteins on insulin metabolism and action.

## 1. General Introduction

Since the discovery of carcinoembryonic antigen (CEA) in 1965 as tumor-specific antigen in human colonic carcinoma, research on this family of proteins has mounted, in particular focusing on one of its members, the carcinoembryonic antigen-related cell adhesion molecule 1 (CEACAM1) plasma membrane glycoprotein [1]. Consistent with its ubiquitous expression and its regulation by metabolic [2,3] as well as immunological factors [4], CEACAM1 exerts several pleiotropic functions that have been well characterized. From the metabolic standpoint, CEACAM1 promotes insulin clearance and mediates a downregulatory effect on fatty acid synthesis by acute insulin pulses [5,6]. It also regulates inflammatory response [7].

CEACAM2 (previously known as Biliary Glycoprotein 2 (Bgp2)) is a close relative plasma membrane glycoprotein of CEACAM1. Consistent with its differential tissue and cell-specific expression [8], CEACAM2 exerts distinct as well as some overlapping functions with CEACAM1. CEACAM2 is involved in spermatid maturation [9,10], platelet activation and adhesion [11] and blood pressure regulation [12]. CEACAM2 is also involved in regulating insulin secretion [13,14] and energy expenditure [15,16]. The role of CEACAM2 in metabolism is the subject of this review.

## 2. Gene Structure of CEACAM2

In contrast to *Ceacam1* gene that is detected in both pre- and postnatal developmental stages [8], *Ceacam2* gene is not expressed during the embryonic stages in mice but starts to appear at three weeks postnatally and its expression continues to increase linearly to adulthood [9].

Mouse *Ceacam2* and *Ceacam1* loci are located on murine chromosome 7 about 62 kb apart [17]. Their genomic sequences share ~79.6% homology [8]. Both genes have nine exons, the seventh undergoes alternative splicing to give rise to an early stop codon resulting in two different transcripts that are distinguished by a long (-L) or a short (-S) intracellular tail, containing or lacking conserved phosphorylation sites, respectively [18,19]. This pair of transcripts contains four IgG-like loops in its extracellular domain (CEACAM-4L/4S).

In addition to exon 7, both exons 3 and 4 undergo alternative splicing to produce two isoforms with two instead of four IgG loops (CEACAM-2L/2S). The incidence of this additional alternative splicing is high in *Ceacam2* and yields a coding domain sequence of 1020 bp encoding CEACAM2-2L and another of 816 bp encoding CEACAM2-2S. Both transcripts share the same N-terminal tail with a signaling peptide and two extracellular loops with one being of V-type IgG and the other of C2-type IgG.

CEACAM2-2L undergoes glycosylation at the extracellular domain to elevate the apparent molecular mass from 37 kDa of apoprotein to 52 kDa. CEACAM2 loses glycosylation as well as the ability to form cis- or trans-polymers after deletion of the first V type IgG domain [10]. The amino acid sequence of the intracellular domains of CEACAM1 and CEACAM2 share ~93% homology, in particular at the putative tyrosine phosphorylation sites at Y_488_ and Y_515_ in CEACAM1-4L and Y_307_, Y_334_ in CEACAM2-2L.

*Ceacam2* transcripts are detected in spleen, testis and prostate [8,17,19,20] and in pooled sorted non-β pancreatic cells [13]. At the protein level, CEACAM2 is expressed in kidneys, uterus, crypt cells and the villi lining the intestinal segment beginning with the distal jejunum, neuroendocrine cells of the ileum and platelets [8,9,11,13,15,17,20]. CEACAM2 protein is also expressed in several neuronal populations in the brain, including the ventromedial hypothalamus (VMH) and other centers involved in feeding behavior and rewards, such as hippocampus, striatum, olfactory bulb, and the globus and ventral pallidus [15]. In contrast, CEACAM2 is virtually absent in tissues that constitute the main sites of insulin action in the periphery (liver, white adipose tissue and skeletal muscle), as opposed to CEACAM1 that is predominantly expressed in the liver and its transcripts are detected in adipose tissue at a minimal level but not in skeletal muscle, under physiologic conditions.

## 3. Role of CEACAM2 in Insulin Secretion

Fluorescence-activated cell sorting of isolated islets revealed a relatively higher level of CEACAM1 expression in pancreatic β-cells [21] as opposed to CEACAM2 that is predominantly expressed in non-β pancreatic cells [13]. Despite its expression in β-cells, global null deletion of *Ceacam1* does not alter glucose-stimulated insulin secretion or β-cell area [21]. In contrast, global deletion of *Ceacam2* causes an increase in β-cell secretory function [13]. This occurs without affecting basal plasma levels of hormones (insulin, glucagon and somatostatin) or without significantly changing the areas of pancreatic cells (α-, β- and δ-cells), as shown by immunohistochemical analysis [13]. Moreover, pooled islets isolated from global *Ceacam2* knockout (*Cc2^−/−^*) mice release normal levels of insulin as compared to islets from wild-type mice in response to both glucose and potassium chloride. Together, this suggests that CEACAM2 regulates insulin secretion via an extra-pancreatic rather than a cell-autonomous regulatory mechanism that directly involves pancreatic cells.

Consistent with CEACAM2 expression in neuroendocrine cells of the distal intestinal villi [13] that secrete the insulinotropic glucagon-like peptide-1 (GLP-1) incretin [22,23,24,25,26,27], studies in mice and in Glutag entero-endocrine cells demonstrated that, CEACAM2 regulates insulin secretion via a mechanism that implicates GLP-1 release [13]. In brief, *Cc2^−/−^* mice exhibit higher excursion of GLP-1 and insulin in response to oral glucose and this effect is blunted by exendin (9–39), a GLP-1 receptor antagonist [13]. Consistently, siRNA-mediated knockdown of *Ceacam2* from cultured Glutag cells showed a significant increase in GLP-1 secretion without affecting proglucagon mRNA levels both basally and in response to glucose [13]. Mechanistically, this appears to implicate increased cellular Ca^2+^ entry via L-type Voltage-gated Ca^2+^ channels, a process that is known to underline GLP-1 release in enterocytes [28,29,30].

In light of its expression in VMH [15], a glucose-sensing center in the brain [31], it is also possible that CEACAM2 regulates insulin secretion primarily via a neuronal-mediated mechanism [32,33]. This possible mechanism remains to be tested.

## 4. Role of CEACAM2 in Insulin Clearance

Insulin metabolism is regulated by insulin secretion from pancreatic β-cells and by its clearance, which occurs mainly in hepatocytes and to a lower extent in renal proximal tubule cells [34]. Upon its pulsatile secretion [35], insulin is rapidly transported via the portal vein into hepatocytes through the fenestrae in the liver sinusoidal endothelium to undergo degradation [36]. In this manner, the liver clears up to 80% of secreted insulin during its first pass [37]. In contrast, insulin transport in extrahepatic insulin target tissues is tightly regulated by endothelial cells [38,39,40], demonstrating a role for these cells in the regulation of peripheral insulin extraction.

Insulin clearance is mediated by receptor-mediated insulin uptake into the cell followed by its degradation in lysosomes as well as in endosomes [41]. Upon its phosphorylation by the insulin receptor tyrosine kinase, CEACAM1 forms a complex with the insulin receptor to increase the rate of insulin uptake and its target to the degradation process in hepatocytes [6] as well as in proximal tubular cells [42]. Several genetically modified loss- and gain-of-function mouse models targeting CEACAM1 in the liver demonstrated a key role for the upregulatory effect of CEACAM1 on receptor-mediated insulin uptake in maintaining insulin sensitivity and limiting de novo lipogenesis in liver in the face of higher insulin levels in the portal than systemic circulation. The role of CEACAM1 in regulating insulin clearance and its underlying mechanism has recently been reviewed [6].

In contrast to CEACAM1, CEACAM2 is not detected to a significant extent in hepatocytes but rather in murine kidney, an important site for insulin extraction. Whether CEACAM2 promotes receptor-mediated insulin uptake in renal proximal tubule cells is currently under investigation. Consistent with the dependence of this function on the phosphorylation of the conserved tyrosine residue in the highly homologous intracellular domain of these related CEACAM membrane glycoproteins [43,44], CEACAM2 is expected to mediate insulin clearance in renal proximal tubular cells. Intact insulin clearance in male *Cc2^−/−^* null mice does not rule out a potential role for CEACAM2 in insulin extraction since it likely results from their intact CEACAM1 expression [15] and its dependent hepatic and renal insulin uptake. However, a potential role for CEACAM2 in extracting endogenously released insulin may not be as critical as that of CEACAM1 given the failure of insulin to regulate its transcription as it does to *Ceacam1* promoter transcriptional activity [2,3,13,45]. In light of the suppressive effect of glucose on *Ceacam2* mRNA levels [13], the role of CEACAM2 in glucose-stimulated insulin secretion is predictably more physiologically significant than its potential role in insulin clearance. 

## 5. Role of CEACAM2 in Food Intake: Effect on Insulin Action

Food intake and energy balance are regulated by leptin-dependent neuronal signals in the arcuate nucleus (ARC) as well as in the dorsomedial (DMH) and ventromedial hypothalamus (VMH) [46]. Using immunohistochemical analysis, we detected CEACAM2 in neuronal hypothalamic populations like VMH, hippocampus, striatum, olfactory bulb and the globus and ventral pallidus [15] that are implicated in the regulation of feeding behavior [15,47,48]. Consistently, both male and female global *Cc2^−/−^* null mice display hyperphagia without changes in plasma leptin levels at its onset. This suggests that hyperphagia in *Cc2^−/−^* mice is not primarily caused by changes in leptin sensitivity [49]. Given that hypothalamic *Ceacam2* mRNA is induced by fasting and reduced upon refeeding in response to glucose release [13,15], it is possible that hyperphagia in *Cc2^−/−^* null mice develops at least in part, from altered glucose sensing activity [31] of the VMH as a consequence of the loss of neuronal CEACAM2.

Hyperinsulinemic-euglycemic clamp analysis demonstrated insulin resistance in skeletal muscle but not in the liver or adipose tissue of *Cc2*^−/−^ females [15], resulting from increased fatty acids uptake followed by incomplete fatty acid β-oxidation and consequently, lipotoxicity [50,51]. Given that CEACAM2 is not expressed in skeletal muscle [8], this points to central dysregulation of insulin action in these mice. Since VMH is a key site of leptin regulation of glucose uptake in skeletal muscle but not white adipose tissue [52], it is conceivable that cellular leptin resistance in *Cc2*^−/−^ females links CEACAM2 to leptin-dependent signaling pathways regulating glucose uptake and energy dissipation [53,54,55]. Thus, it is possible that peripheral insulin resistance in *Cc2*^−/−^ females is caused, at least in part, by altered leptin-dependent signaling pathways in VMH regulating glucose disposal and energy dissipation [54,56].

Young *Cc2^−/−^* males exhibit increased fatty acid uptake in skeletal muscle and in the mitochondria to undergo complete fatty acid β-oxidation. This led to insulin sensitivity and lower total fat mass. With age, fat mass and visceral adiposity increase, while the metabolically active lean mass decreases in parallel to reduced glucose uptake in skeletal muscle that constitutes a main site of energy expenditure [57]. Since CEACAM2 is not expressed in skeletal muscle [8], the progressive age-related decline in energy dissipation in *Cc2^−/−^* males likely stems from central dysregulation of peripheral glucose disposal, as is the case for their female counterparts [15]. Given that hypothalamic *Ceacam2* mRNA level remains intact with age, unlike that of *Ceacam1* that declines progressively until it reaches a loss by >70% at nine months of age to contribute to hyperphagia and disturb energy balance [58], it is likely that reduced *Ceacam1* mRNA amplifies the adverse effect of *Ceacam2* deletion on the hypothalamic control of glucose disposal and energy expenditure in older *Cc2^−/−^* males.

Moreover, at this older age, male mutants develop insulin resistance in liver in addition to skeletal muscle [14]. The hepatic insulin resistance likely results from impaired CEACAM1-dependent hepatic insulin clearance pathways and resultant chronic hyperinsulinemia [14]. The progressive decrease in hepatic *Ceacam1* mRNA stems from a compromised ability of insulin to induce *Ceacam1* transcription under conditions of hyperphagia-driven insulin resistance [2,3] and from PPARα activation by lipolysis-derived fatty acids [3]. Reduced hepatic CEACAM1 levels provide a positive feedback mechanism on fatty acid β-oxidation [3] to prevent hepatic steatosis in aged *Cc2^−/−^* males and to produce acetyl-CoA with the overarching goal to prevent glycolysis and reroute pyruvate to gluconeogenesis and glucose-6-phosphate to the glycogen synthetic pathways [3,14,59]. This is consistent with a role for reduced hepatic CEACAM1 levels in limiting fasting hyperglycemia [60] that could result from excessive increase in insulin secretion in aged *Cc2^−/−^* males.

Pair-feeding experiments show that hyperphagia causes insulin resistance in female and male *Cc2^−/−^* mutants at two and nine months of age, respectively [14,15]. Subsequently, *Cc2^−/−^* mutants develop compromised energy expenditure and reduced locomotor activity [14,15]. The resulting energy imbalance leads to increase in body weight and visceral obesity at about six months of age in females [15] and at about nine months of age in males [14]. In contrast to female mice, young *Cc2^−/−^* males exhibit increased sympathetic tone to white adipose tissue, consistent with induced brown adipogenesis in this depot and higher energy dissipation [16,61]. With age, the surrogate markers of brown adipogenesis (*Ucp1* and *Dio2)* [62] and activated sympathetic tone (*Ucp1, Adβ2r* and *Adβ3r*) [63] are progressively reduced in white adipose tissue, consistent with reduced energy expenditure and increased visceral obesity in older *Cc2^−/−^* males [14]. This age-related disturbance in energy dissipation could result, at least partially, from a loss of CEACAM2 at the VMH that contributes significantly to the central regulation of energy balance [64,65]. Thus, the hypermetabolic state (manifested by complete β-oxidation in skeletal muscle, increased brown adipogenesis in brown and white adipose depots, and increased sympathetic tone to adipose tissue), appears to offset the negative effect of hyperphagia in young *Cc2^−/−^* males and maintain them insulin-sensitive until 8–9 months of age when they become hypometabolic exhibiting lower spontaneous physical activity than their wild-type counterparts and developing systemic insulin resistance [14,16].

Hyperphagia can also result from chronic hyperinsulinemia and insulin resistance [66,67,68,69], which develops in *Cc2^−/−^* females at ~2 months of age arising chiefly from increased insulin secretion [13,15]. In males, the persistent increase in insulin secretion, in part mediated by the higher plasma GLP-1 secretion [13], is offset by a parallel increase in CEACAM1-mediated insulin clearance, resulting in normoinsulinemia in the young until ~9 months of age when chronic hyperinsulinemia develops largely from impaired hepatic insulin clearance that fails to counter the sustained elevation in insulin secretion [14]. Impaired insulin extraction in older males, results from the age-related progressive decline in hepatic CEACAM1 levels [14,58]. Nonetheless, hyperinsulinemia induces the transcriptional activity of SREBP-1c to stimulate the expression of lipogenic genes [70], such as fatty acid synthase (FASN), followed by their activation. Since the rise of hypothalamic FASN activity mediates hyperphagia independently of leptin [71,72,73], it is likely that hyperphagia is sustained by hyperinsulinemia-driven increase in hypothalamic FASN activity in *Cc2^−/−^* mutants [14,15]. Additionally, elevated hypothalamic FASN activity could contribute to dysregulated central control of peripheral glucose disposal and reduced fatty acid β-oxidation in skeletal muscle of *Cc2*^−/−^ females and *Cc2*^−/−^ males at ≥9 months of age [14,15,72].

Hyperinsulinemia can also induce FASN activity in the liver. With the progressive decrease of hepatic CEACAM1 expression as *Cc2*^−/−^ males age, the counterregulatory CEACAM1-dependent negative effect of insulin on hepatic FASN activity [74] is abolished, giving rise to excessive lipid formation and re-esterification in the liver, followed by its redistribution to the white adipose depot for storage and subsequently, visceral obesity [58]. The resultant increase in lipolysis [58] as well as the pro-inflammatory state [75] contribute to systemic insulin resistance that develops in *Cc2*^−/−^ males at ≥9 months of age [14,15].

## 6. Conclusions

**Figure 1:**
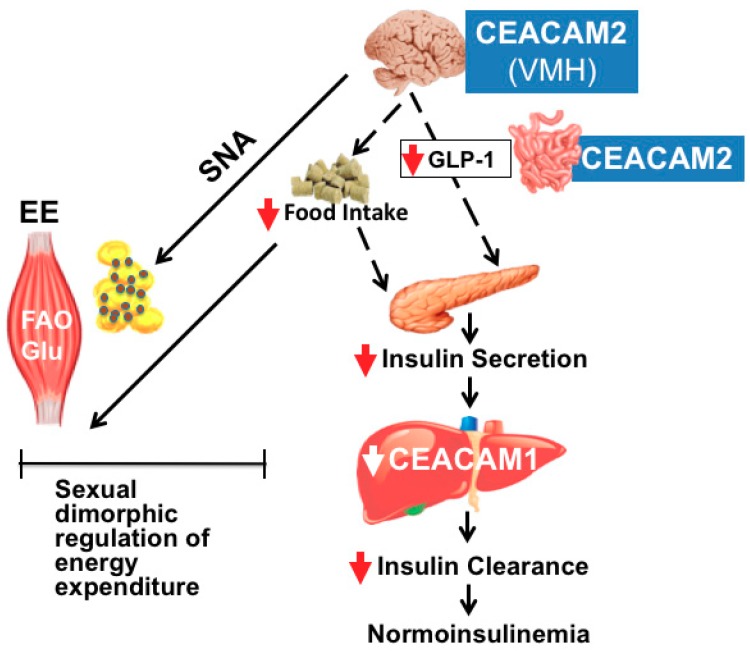
Metabolic role of CEACAM2. SNA, sympathetic nervous activity; EE, energy expenditure; FAO, fatty acid β-oxidation; Glu, glucose.

Based on the phenotype of *Cc2^−/−^* mice, we propose that at fed state, when glucose is released, CEACAM2 expression rapidly declines in the entero-endocrine cells (as well as the neuroendocrine cells of the hypothalamus) [15] to stimulate insulin secretion via GLP-1–dependent mechanisms (Graphical Abstract). This in turn, induces CEACAM1–dependent hepatic insulin clearance [2,21,44] to maintain normoinsulinemia and insulin sensitivity. Given that GLP-1 prompts transition into the fasting state [76], this may initiate a negative feedback mechanism to recover hypothalamic CEACAM2 expression and subsequently, limit food intake and insulin secretion (Graphical Abstract). Further studies are needed to decipher the mechanisms underlying the role of CEACAM2 in controlling food intake but our data show that both leptin-dependent and leptin-independent hypothalamic pathways are implicated. Nonetheless, involvement of CEACAM2 in the central regulation of feeding behavior in addition to energy dissipation in skeletal muscle and insulin secretion is consistent with its expression in VMH, which contributes to the central regulation of energy balance and glucose disposal via sympathetic relay to peripheral tissues [65,77,78]. Moreover, the observed sexual dimorphism in obesity in *Cc2^−/−^* null mutants further links CEACAM2 to the regulation of obesity and insulin resistance by VMH since lesions in this neuronal population cause obesity more commonly in female than male rodents [65]. The phenotype of *Cc2^−/−^* mice provides an in vivo demonstration that CEACAM2 in the neuroendocrine cells of ileum and hypothalamus downregulates insulin secretion by suppressing GLP-1 release in male and female mice (Figure 1). Becasuse insulin upregulates hepatic CEACAM1 expression [2,3], the decrease in insulin secretion by CEACAM2 lowers hepatic CEACAM1 expression to limit insulin clearance and maintain normoinsulinemia in the face of restricted insulin secretion. CEACAM2 also limits food intake in both males and females but its deletion causes a reduction in sympathetic nervous activity in females only. This sexual dimorphism in terms of energy expenditure causes sex-dependent regulation of insulin action with *Cc2^−/−^* females developing insulin resistance and *Cc2^−/−^* males developing insulin sensitivity until about 8-9 months of age.

The progression of insulin resistance in age-dependent manner in *Cc2^−/−^* males [14] appears to involve the differential reduction of CEACAM1 in the hypothalamus [58] as well as in the liver [14]. The former contributes to leptin resistance and reduced spontaneous physical activity [79] and the latter to hyperinsulinemia-driven energy imbalance and systemic insulin resistance, at least partly by blunting hepatic insulin action [58,80]. This links insulin clearance to insulin secretion in the overall systemic regulation of physiologic insulin levels and provides further evidence for the impact of the coordinated regulatory effect of CEACAM proteins in insulin metabolism and action. 

## References

[1] Horst A.K., Najjar S.M., Wagener C., Tiegs G. (2018). Ceacam1 in liver injury, metabolic and immune regulation. Int. J. Mol. Sci..

[2] Najjar S., Boisclair Y., Nabih Z., Philippe N., Imai Y., Suzuki Y., Suh D., Ooi G. (1996). Cloning and characterization of a functional promoter of the rat pp120 gene, encoding a substrate of the insulin receptor tyrosine kinase. J. Biol. Chem..

[3] Ramakrishnan S.K., Khuder S.S., Al-Share Q.Y., Russo L., Abdallah S.L., Patel P.R., Heinrich G., Muturi H.T., Mopidevi B.R., Oyarce A.M. (2016). Pparalpha (peroxisome proliferator-activated receptor alpha) activation reduces hepatic ceacam1 protein expression to regulate fatty acid oxidation during fasting-refeeding transition. J. Biol. Chem..

[4] Dery K.J., Silver C., Yang L., Shively J.E. (2018). Interferon regulatory factor 1 and a variant of heterogeneous nuclear ribonucleoprotein l coordinately silence the gene for adhesion protein ceacam1. J. Biol. Chem..

[5] Heinrich G., Ghadieh H.E., Ghanem S.S., Muturi H.T., Rezaei K., Al-Share Q.Y., Bowman T.A., Zhang D., Garofalo R.S., Yin L. (2017). Loss of hepatic ceacam1: A unifying mechanism linking insulin resistance to obesity and non-alcoholic fatty liver disease. Front. Endocrinol. (Lausanne).

[6] Najjar S.M., Perdomo G. (2019). Hepatic insulin clearance: Mechanism and physiology. Physiology (Bethesda).

[7] Gray-Owen S.D., Blumberg R.S. (2006). Ceacam1: Contact-dependent control of immunity. Nat. Rev. Immunol..

[8] Han E., Phan D., Lo P., Poy M.N., Behringer R., Najjar S.M., Lin S.H. (2001). Differences in tissue-specific and embryonic expression of mouse ceacam1 and ceacam2 genes. Biochem. J..

[9] Salaheldeen E., Kurio H., Howida A., Iida H. (2012). Molecular cloning and localization of a ceacam2 isoform, ceacam2-l, expressed in spermatids in mouse testis. Mol. Reprod. Dev..

[10] Salaheldeen E., Howida A., Wakayama T., Iida H. (2014). Ceacam2-l on spermatids interacts with poliovirus receptor on sertoli cells in mouse seminiferous epithelium. J. Histochem. Cytochem..

[11] Alshahrani M.M., Yang E., Yip J., Ghanem S.S., Abdallah S.L., deAngelis A.M., O’Malley C.J., Moheimani F., Najjar S.M., Jackson D.E. (2014). Ceacam2 negatively regulates hemi (itam-bearing) gpvi and clec-2 pathways and thrombus growth in vitro and in vivo. Blood.

[12] Gupta S., Yan Y., Malhotra D., Liu J., Xie Z., Najjar S.M., Shapiro J.I. (2012). Ouabain and insulin induce sodium pump endocytosis in renal epithelium. Hypertension.

[13] Ghanem S.S., Heinrich G., Lester S.G., Pfeiffer V., Bhattacharya S., Patel P.R., DeAngelis A.M., Dai T., Ramakrishnan S.K., Smiley Z.N. (2016). Increased glucose-induced secretion of glucagon-like peptide-1 in mice lacking the carcinoembryonic antigen-related cell adhesion molecule 2 (ceacam2). J. Biol. Chem..

[14] Ghanem S.S., Muturi H.T., DeAngelis A.M., Hu J., Kulkarni R.N., Heinrich G., Najjar S.M. (2017). Age-dependent insulin resistance in male mice with null deletion of the carcinoembryonic antigen-related cell adhesion molecule 2 gene. Diabetologia.

[15] Heinrich G., Ghosh S., Deangelis A.M., Schroeder-Gloeckler J.M., Patel P.R., Castaneda T.R., Jeffers S., Lee A.D., Jung D.Y., Zhang Z. (2010). Carcinoembryonic antigen-related cell adhesion molecule 2 controls energy balance and peripheral insulin action in mice. Gastroenterology.

[16] Patel P.R., Ramakrishnan S.K., Kaw M.K., Raphael C.K., Ghosh S., Marino J.S., Heinrich G., Lee S.J., Bourey R.E., Hill J.W. (2012). Increased metabolic rate and insulin sensitivity in male mice lacking the carcinoembryonic antigen-related cell adhesion molecule 2. Diabetologia.

[17] Nedellec P., Dveksler G.S., Daniels E., Turbide C., Chow B., Basile A.A., Holmes K.V., Beauchemin N. (1994). Bgp2, a new member of the carcinoembryonic antigen-related gene family, encodes an alternative receptor for mouse hepatitis viruses. J. Virol..

[18] Najjar S.M., Accili D., Philippe N., Jernberg J., Margolis R., Taylor S.I. (1993). Pp120/ecto-atpase, an endogenous substrate of the insulin receptor tyrosine kinase, is expressed as two variably spliced isoforms. J. Biol. Chem..

[19] Robitaille J., Izzi L., Daniels E., Zelus B., Holmes K.V., Beauchemin N. (1999). Comparison of expression patterns and cell adhesion properties of the mouse biliary glycoproteins bbgp1 and bbgp2. Eur. J. Biochem..

[20] Zebhauser R., Kammerer R., Eisenried A., McLellan A., Moore T., Zimmermann W. (2005). Identification of a novel group of evolutionarily conserved members within the rapidly diverging murine cea family. Genomics.

[21] DeAngelis A.M., Heinrich G., Dai T., Bowman T.A., Patel P.R., Lee S.J., Hong E.G., Jung D.Y., Assmann A., Kulkarni R.N. (2008). Carcinoembryonic antigen-related cell adhesion molecule 1: A link between insulin and lipid metabolism. Diabetes.

[22] D’Alessio D. (2016). Is glp-1 a hormone: Whether and when?. J. Diabetes Investig..

[23] Reimann F., Gribble F.M. (2002). Glucose-sensing in glucagon-like peptide-1-secreting cells. Diabetes.

[24] Kedees M.H., Guz Y., Grigoryan M., Teitelman G. (2013). Functional activity of murine intestinal mucosal cells is regulated by the glucagon-like peptide-1 receptor. Peptides.

[25] Habib A.M., Richards P., Rogers G.J., Reimann F., Gribble F.M. (2013). Co-localisation and secretion of glucagon-like peptide 1 and peptide yy from primary cultured human l cells. Diabetologia.

[26] Seino Y., Yabe D. (2013). Glucose-dependent insulinotropic polypeptide and glucagon-like peptide-1: Incretin actions beyond the pancreas. J. Diabetes Investig..

[27] Drucker D.J. (2015). Deciphering metabolic messages from the gut drives therapeutic innovation: The 2014 banting lecture. Diabetes.

[28] Sidhu S.S., Thompson D.G., Warhurst G., Case R.M., Benson R.S. (2000). Fatty acid-induced cholecystokinin secretion and changes in intracellular ca2+ in two enteroendocrine cell lines, stc-1 and glutag. J. Physiol..

[29] Reimann F., Maziarz M., Flock G., Habib A.M., Drucker D.J., Gribble F.M. (2005). Characterization and functional role of voltage gated cation conductances in the glucagon-like peptide-1 secreting glutag cell line. J. Physiol..

[30] Nadkarni P., Chepurny O.G., Holz G.G. (2014). Regulation of glucose homeostasis by glp-1. Prog. Mol. Biol. Transl. Sci..

[31] Levin B.E., Routh V.H., Kang L., Sanders N.M., Dunn-Meynell A.A. (2004). Neuronal glucosensing: What do we know after 50 years?. Diabetes.

[32] Thorens B. (2010). Central control of glucose homeostasis: The brain--endocrine pancreas axis. Diabetes Metab..

[33] Osundiji M.A., Lam D.D., Shaw J., Yueh C.Y., Markkula S.P., Hurst P., Colliva C., Roda A., Heisler L.K., Evans M.L. (2012). Brain glucose sensors play a significant role in the regulation of pancreatic glucose-stimulated insulin secretion. Diabetes.

[34] Polidori D.C., Bergman R.N., Chung S.T., Sumner A.E. (2016). Hepatic and extrahepatic insulin clearance are differentially regulated: Results from a novel model-based analysis of intravenous glucose tolerance data. Diabetes.

[35] Satin L.S., Butler P.C., Ha J., Sherman A.S. (2015). Pulsatile insulin secretion, impaired glucose tolerance and type 2 diabetes. Mol. Asp. Med..

[36] Mohamad M., Mitchell S.J., Wu L.E., White M.Y., Cordwell S.J., Mach J., Solon-Biet S.M., Boyer D., Nines D., Das A. (2016). Ultrastructure of the liver microcirculation influences hepatic and systemic insulin activity and provides a mechanism for age-related insulin resistance. Aging Cell.

[37] Duckworth W.C., Bennett R.G., Hamel F.G. (1998). Insulin degradation: Progress and potential. Endocrinol. Rev..

[38] Barrett E.J., Eggleston E.M., Inyard A.C., Wang H., Li G., Chai W., Liu Z. (2009). The vascular actions of insulin control its delivery to muscle and regulate the rate-limiting step in skeletal muscle insulin action. Diabetologia.

[39] Kolka C.M., Bergman R.N. (2012). The barrier within: Endothelial transport of hormones. Physiology (Bethesda).

[40] Lee W.L., Klip A. (2016). Endothelial transcytosis of insulin: Does it contribute to insulin resistance?. Physiology (Bethesda).

[41] Tokarz V.L., MacDonald P.E., Klip A. (2018). The cell biology of systemic insulin function. J. Cell Biol..

[42] Al-Share Q.Y., DeAngelis A.M., Lester S.G., Bowman T.A., Ramakrishnan S.K., Abdallah S.L., Russo L., Patel P.R., Kaw M.K., Raphael C.K. (2015). Forced hepatic overexpression of ceacam1 curtails diet-induced insulin resistance. Diabetes.

[43] Formisano P., Najjar S.M., Gross C.N., Philippe N., Oriente F., Kern-Buell C.L., Accili D., Gorden P. (1995). Receptor-mediated internalization of insulin. Potential role of pp120/ha4, a substrate of the insulin receptor kinase. J. Biol. Chem..

[44] Poy M.N., Yang Y., Rezaei K., Fernstrom M.A., Lee A.D., Kido Y., Erickson S.K., Najjar S.M. (2002). Ceacam1 regulates insulin clearance in liver. Nat. Genet..

[45] Ghadieh H.E., Muturi H.T., Najjar S.M. (2017). Exenatide prevents diet-induced hepatocellular injury in a ceacam1-dependent mechanism. J. Diabetes Treat..

[46] Pan W.W., Myers M.G. (2018). Leptin and the maintenance of elevated body weight. Nat. Rev. Neurosci..

[47] Morton G.J., Cummings D.E., Baskin D.G., Barsh G.S., Schwartz M.W. (2006). Central nervous system control of food intake and body weight. Nature.

[48] Cota D., Barrera J.G., Seeley R.J. (2006). Leptin in energy balance and reward: Two faces of the same coin?. Neuron.

[49] Bingham N.C., Anderson K.K., Reuter A.L., Stallings N.R., Parker K.L. (2008). Selective loss of leptin receptors in the ventromedial hypothalamic nucleus results in increased adiposity and a metabolic syndrome. Endocrinology.

[50] Wang H., Knaub L.A., Jensen D.R., Young Jung D., Hong E.G., Ko H.J., Coates A.M., Goldberg I.J., de la Houssaye B.A., Janssen R.C. (2009). Skeletal muscle-specific deletion of lipoprotein lipase enhances insulin signaling in skeletal muscle but causes insulin resistance in liver and other tissues. Diabetes.

[51] Koves T.R., Ussher J.R., Noland R.C., Slentz D., Mosedale M., Ilkayeva O., Bain J., Stevens R., Dyck J.R., Newgard C.B. (2008). Mitochondrial overload and incomplete fatty acid oxidation contribute to skeletal muscle insulin resistance. Cell Metab..

[52] Toda C., Shiuchi T., Lee S., Yamato-Esaki M., Fujino Y., Suzuki A., Okamoto S., Minokoshi Y. (2009). Distinct effects of leptin and a melanocortin receptor agonist injected into medial hypothalamic nuclei on glucose uptake in peripheral tissues. Diabetes.

[53] Schwartz M.W., Woods S.C., Porte D., Seeley R.J., Baskin D.G. (2000). Central nervous system control of food intake. Nature.

[54] Badman M.K., Flier J.S. (2007). The adipocyte as an active participant in energy balance and metabolism. Gastroenterology.

[55] Flak J.N., Myers M.G. (2016). Minireview: Cns mechanisms of leptin action. Mol. Endocrinol..

[56] Hinoi E., Gao N., Jung D.Y., Yadav V., Yoshizawa T., Kajimura D., Myers M.G., Chua S.C., Wang Q., Kim J.K. (2009). An osteoblast-dependent mechanism contributes to the leptin regulation of insulin secretion. Ann N. Y. Acad Sci.

[57] Yalamanchi S.V., Stewart K.J., Ji N., Golden S.H., Dobs A., Becker D.M., Vaidya D., Kral B.G., Kalyani R.R. (2016). The relationship of fasting hyperglycemia to changes in fat and muscle mass after exercise training in type 2 diabetes. Diabetes Res. Clin. Pract..

[58] Ghadieh H.E., Russo L., Muturi H.T., Ghanem S.S., Manaserh I.H., Noh H.L., Suk S., Kim J.K., Hill J.W., Najjar S.M. (2019). Hyperinsulinemia drives hepatic insulin resistance in male mice with liver-specific ceacam1 deletion independently of lipolysis. Metabolism.

[59] Hue L., Taegtmeyer H. (2009). The randle cycle revisited: A new head for an old hat. Am. J. Physiol. Endocrinol. Metab..

[60] Titchenell P.M., Quinn W.J., Lu M., Chu Q., Lu W., Li C., Chen H., Monks B.R., Chen J., Rabinowitz J.D. (2016). Direct hepatocyte insulin signaling is required for lipogenesis but is dispensable for the suppression of glucose production. Cell Metab..

[61] Rosenbaum M., Leibel R.L. (2010). Adaptive thermogenesis in humans. Int. J. Obes..

[62] de Jesus L.A., Carvalho S.D., Ribeiro M.O., Schneider M., Kim S.W., Harney J.W., Larsen P.R., Bianco A.C. (2001). The type 2 iodothyronine deiodinase is essential for adaptive thermogenesis in brown adipose tissue. J. Clin. Investig..

[63] Plum L., Rother E., Munzberg H., Wunderlich F.T., Morgan D.A., Hampel B., Shanabrough M., Janoschek R., Konner A.C., Alber J. (2007). Enhanced leptin-stimulated pi3k activation in the cns promotes white adipose tissue transdifferentiation. Cell Metab..

[64] Saito M., Minokoshi Y., Shimazu T. (1985). Brown adipose tissue after ventromedial hypothalamic lesions in rats. Am. J. Physiol. Endocrinol. Metab..

[65] King B.M. (2006). The rise, fall, and resurrection of the ventromedial hypothalamus in the regulation of feeding behavior and body weight. Physiol. Behav..

[66] Obici S., Feng Z., Karkanias G., Baskin D.G., Rossetti L. (2002). Decreasing hypothalamic insulin receptors causes hyperphagia and insulin resistance in rats. Nat. Neurosci..

[67] Shin A.C., Filatova N., Lindtner C., Chi T., Degann S., Oberlin D., Buettner C. (2017). Insulin receptor signaling in pomc, but not agrp, neurons controls adipose tissue insulin action. Diabetes.

[68] Plum L., Schubert M., Bruning J.C. (2005). The role of insulin receptor signaling in the brain. Trends Endocrinol. Metab..

[69] Erion K.A., Corkey B.E. (2017). Hyperinsulinemia: A cause of obesity?. Curr. Obes. Rep..

[70] Horton J.D., Goldstein J.L., Brown M.S. (2002). Srebps: Activators of the complete program of cholesterol and fatty acid synthesis in the liver. J. Clin. Investig..

[71] Loftus T.M., Jaworsky D.E., Frehywot G.L., Townsend C.A., Ronnett G.V., Lane M.D., Kuhajda F.P. (2000). Reduced food intake and body weight in mice treated with fatty acid synthase inhibitors. Science.

[72] Cha S.H., Hu Z., Chohnan S., Lane M.D. (2005). Inhibition of hypothalamic fatty acid synthase triggers rapid activation of fatty acid oxidation in skeletal muscle. Proc. Natl. Acad. Sci. USA.

[73] Chakravarthy M.V., Zhu Y., Lopez M., Yin L., Wozniak D.F., Coleman T., Hu Z., Wolfgang M., Vidal-Puig A., Lane M.D. (2007). Brain fatty acid synthase activates pparalpha to maintain energy homeostasis. J.Clin. Investig..

[74] Najjar S.M., Yang Y., Fernstrom M.A., Lee S.J., Deangelis A.M., Rjaily G.A., Al-Share Q.Y., Dai T., Miller T.A., Ratnam S. (2005). Insulin acutely decreases hepatic fatty acid synthase activity. Cell Metab..

[75] Najjar S.M., Russo L. (2014). Ceacam1 loss links inflammation to insulin resistance in obesity and non-alcoholic steatohepatitis (nash). Semin. Immunopathol..

[76] Barrera J.G., Sandoval D.A., D’Alessio D.A., Seeley R.J. (2011). Glp-1 and energy balance: An integrated model of short-term and long-term control. Nat. Rev. Endocrinol..

[77] Haque M.S., Minokoshi Y., Hamai M., Iwai M., Horiuchi M., Shimazu T. (1999). Role of the sympathetic nervous system and insulin in enhancing glucose uptake in peripheral tissues after intrahypothalamic injection of leptin in rats. Diabetes.

[78] Shiuchi T., Haque M.S., Okamoto S., Inoue T., Kageyama H., Lee S., Toda C., Suzuki A., Bachman E.S., Kim Y.B. (2009). Hypothalamic orexin stimulates feeding-associated glucose utilization in skeletal muscle via sympathetic nervous system. Cell Metab..

[79] Heinrich G., Russo L., Castaneda T.R., Pfeiffer V., Ghadieh H.E., Ghanem S.S., Wu J., Faulkner L.D., Ergun S., McInerney M.F. (2016). Leptin resistance contributes to obesity in mice with null mutation of carcinoembryonic antigen-related cell adhesion molecule 1. J. Biol. Chem..

[80] Russo L., Muturi H.T., Ghadieh H.E., Ghanem S.S., Bowman T.A., Noh H.L., Dagdeviren S., Dogbey G.Y., Kim J.K., Heinrich G. (2017). Liver-specific reconstitution of ceacam1 reverses the metabolic abnormalities caused by its global deletion in male mice. Diabetologia.

